# AFLP Polymorphisms Allow High Resolution Genetic Analysis of American Tegumentary Leishmaniasis Agents Circulating in Panama and Other Members of the *Leishmania* Genus

**DOI:** 10.1371/journal.pone.0073177

**Published:** 2013-09-09

**Authors:** Carlos M. Restrepo, Carolina De La Guardia, Octavio E. Sousa, José E. Calzada, Patricia L. Fernández, Ricardo Lleonart

**Affiliations:** 1 Instituto de Investigaciones Científicas y Servicios de Alta Tecnología (INDICASAT-AIP), Ciudad de Panamá, Panamá; 2 Centro de Investigaciones y Diagnóstico de Enfermedades Parasitarias (CIDEP), Facultad de Medicina, Universidad de Panamá, Ciudad de Panamá, Panamá; 3 Instituto Conmemorativo Gorgas de Estudios de la Salud, Ciudad de Panamá, Panamá; 4 Department of Biotechnology, Acharya Nagarjuna University, Guntur, India; Technion-Israel Institute of Technology, Israel

## Abstract

American Tegumentary Leishmaniasis is caused by parasites of the genus *Leishmania*, and causes significant health problems throughout the Americas. In Panama, *Leishmania* parasites are endemic, causing thousands of new cases every year, mostly of the cutaneous form. In the last years, the burden of the disease has increased, coincident with increasing disturbances in its natural sylvatic environments. The study of genetic variation in parasites is important for a better understanding of the biology, population genetics, and ultimately the evolution and epidemiology of these organisms. Very few attempts have been made to characterize genetic polymorphisms of parasites isolated from Panamanian patients of cutaneous leishmaniasis. Here we present data on the genetic variability of local isolates of *Leishmania,* as well as specimens from several other species, by means of Amplified Fragment Length Polymorphisms (AFLP), a technique seldom used to study genetic makeup of parasites. We demonstrate that this technique allows detection of very high levels of genetic variability in local isolates of *Leishmania panamensis* in a highly reproducible manner. The analysis of AFLP fingerprints generated by unique selective primer combinations in *L. panamensis* suggests a predominant clonal mode of reproduction. Using fluorescently labeled primers, many taxon-specific fragments were identified which may show potential as species diagnostic fragments. The AFLP permitted a high resolution genetic analysis of the *Leishmania* genus, clearly separating certain groups among *L. panamensis* specimens and highly related species such as *L. panamensis* and *L. guyanensis*. The phylogenetic networks reconstructed from our AFLP data are congruent with established taxonomy for the genus *Leishmania*, even when using single selective primer combinations. Results of this study demonstrate that AFLP polymorphisms can be informative for genetic characterization in *Leishmania* parasites, at both intra and inter-specific levels.

## Introduction

Leishmaniasis is a neglected tropical disease caused by protozoa of the genus *Leishmania*, and has a variety of clinical manifestations, ranging from the mild cutaneous to life threatening visceral leishmaniasis [1]. In Panama, cutaneous and muco-cutaneous leishmaniasis are the main manifestations, caused almost exclusively by *Leishmania panamensis* [2]–[5]. Although some authors have sporadically reported the presence of other species, their clinical relevance has not been demonstrated [5]–[11]. The burden of the disease in the country is on the rise, as growing urban areas get in closer relationship with sylvatic reservoirs [2], [5], [12].

The study of the genetic diversity in *Leishmania* is very important in order to unveil key aspects of the population genetics and epidemiology of this parasite. The genetic variability of *Leishmania* parasites has been studied using various genetic marker systems, including both protein based multilocus enzyme electrophoresis (MLEE, reviewed by [13]), and DNA based polymorphism detection tools (PCR – RFLP or DNA sequence typing). Although MLEE has been very useful in the past to study genetic variation in *Leishmania*, it has important limitations, leading researchers to explore and evaluate DNA sequence based systems as potentially more user-friendly and efficient. Some of the DNA loci studied in *Leishmania* include the ribosomal internal transcribed spacer (ITS, [14]), gp63 genes [15], and hsp70 genes [16]. These studies have covered several *Leishmania* species from both Old and New World territories. However, data on the genetic composition of *Leishmania* species that cause the disease in Panama are scarce and are necessary for a better understanding of key aspects of the biology, genetics and epidemiology of the parasite. Although some attempts have been made previously to characterize the genetic diversity of the *Leishmania* species causing leishmaniasis in Panama, particularly using kinetoplast based RFLP [17] and ITS sequencing [18], more data are required for a better understanding of the local parasite populations.

Here we have explored amplified fragment length polymorphisms (AFLP) to characterize genetic variability of *Leishmania* parasites isolated from Panamanian cutaneous Leishmaniasis patients. AFLP is a very useful technique for rapidly visualizing polymorphic DNA fragments from organisms with no previous sequence information [19]. This technique has been shown to be highly reproducible as it combines the specificity of restriction fragment length polymorphisms (RFLP) with the sensitivity of the polymerase chain reaction (PCR). AFLP has been successfully used to study the biology, genetics, ecology, and phylogeny of many organisms [20]. The use of AFLP in *Leishmania* parasites has been limited, with only a few reports in members of the *Leishmania* (*Leishmania*) subgenus [21]–[24]. Here we probed the reproducibility and suitability of the technique for scanning the genome of *Leishmania* parasites for polymorphisms. We demonstrate that AFLP is very useful for both intra and inter-species genetic analyses.

## Materials and Methods

### Ethics Statement

This research was approved by the INDICASAT-AIP institutional review board. Although data were analyzed anonymously, written informed consent was obtained from patients before the samples were taken.

### Parasites and Culture

*Leishmania* isolates were obtained from biopsy samples of cutaneous leishmaniasis patients in Panama City and other parts of Panama Province. Reference strains were obtained from several sources, including cryobanks at INDICASAT-AIP, University of Panama, Instituto Conmemorativo Gorgas de Estudios de la Salud, Walter Reed Army and the *Leishmania* collection at the Instituto Osvaldo Cruz (CLIOC) (Table 1). Primary parasite isolations were done in NNN biphasic medium [25] at room temperature. Promastigotes were then cultured at 25°C in T25 tissue culture flasks containing 10 ml of Schneider’s insect medium (Sigma, USA) plus gentamycin (50 µg/ml) and 20% (v/v) heat-inactivated fetal calf serum.

**Table 1:** *Leishmania* specimens used in this study.

Species	Code	Source and characteristics
*L. (V.) panamensis*	Ps	Reference strain, Centro de Investigación y Diagnóstico de Enfermedades Parasitarias, Facultad de Medicina, Universidad de Panamá (CIDEP)
*L. (V.) panamensis*	P1 to P26	Field isolates from *Leishmania* specimen bank, INDICASAT-AIP
*L. (L.) chagasi*	Cha2	Reference strain, CIDEP
*L. (L.) chagasi*	Cha5	Reference strain, CIDEP
*L. (L.) mexicana*	Mex	*Leishmania* Type Culture Collection, Instituto Oswaldo Cruz, IOC-L 561, International Reference MHOM/BZ/1982/BEL21
*L. (L.) aristidesi*	Ari	Reference strain, CIDEP
*L. (L.) major*	Maj	Strain donated by Dr. M. Bozza, Federal University of Rio de Janeiro.
*L. (L.) major*	Majw1	MHOM/SA/1991/WR-1088 (^1^)
*L. (V.) lainsoni*	Lai	IOC-L 1023, MHOM/BR/1981/M6426
*L. (V.) braziliensis*	Bra	IOC-L 566, MHOM/BR/1975/M2903
*L. (V.) guyanensis*	Gc	Reference strain, MHOM/BR/1975/M4147
*L. (V.) guyanensis*	Gf	Reference strain Dr. M. Bozza, Federal University of Rio de Janeiro, IOC/L 0565
*L. (V.) guyanensis*	Gw3	MHOM/GF/2010/WR-3017 (^1^)
*L. (L.) donovani*	Doni	MHOM/SD/75/1246 Kartown (^1^)
*L. (L.) donovani*	Donw2	MHOM/IN/2006/WR-2801 (^1^)
*L. (L.) donovani*	Donw3	MHOM/SD/1980/Hansen-WR-378 (^1^)
*L. (V.) peruviana*	Perw2	MHOM/PE/2005/WR-2771 (^1^)

(^1^) Kindly provided by Dr. C. Spadafora.

### DNA Extractions

High molecular weight DNA was extracted from stationary phase promastigote cultures using a salting out procedure as recommended by the manufacturer (Wizard® Genomic DNA purification kit, Promega, USA). DNA aliquots were checked for integrity, digestibility, and absence of nucleases or PCR inhibitors. Species identities were verified by *hsp70* PCR – RFLP, using MluI, RsaI, BccI or HaeIII restriction endonucleases [16], [26].

### AFLP

Fluorescent AFLP reactions were done using a commercial kit, as recommended by the manufacturer (AFLP Microbial Fingerprinting Kit, P/N 402948, Applied Biosystems, USA). Based on standard primer combination scanning procedures previously performed on a small number of *L. panamensis* isolates [27], thirteen selective primer combinations were chosen and used at the selective amplification step. Amplification products were detected on an automated sequencer Genetic Analyzer 3130 using GeneScan ROX 500 as internal size standard (Applied Biosystems, USA). Electropherograms were analyzed using GeneMarker software v2.2.0 (SoftGenetics LLC, USA) using the default parameters recommended as optimal for AFLP markers by the manufacturer (http://www.softgenetics.com/GeneMarker.html). Further details of AFLP protocol and parameters used for allele calling are included in Supporting Information. Peak patterns were converted to dominant presence - absence (1-0) matrices. Measures were taken to minimize scoring errors, which included careful examination of each electropherogram to exclude doubtful peaks, manual checking of bin sets defined by the software, setting minimal threshold at 100 relative fluorescent units, and considering only peaks with sizes between 50 and 500 base pairs. Although some preliminary analyses were done on individual matrices (matrices generated by one selective primer combination), a single concatenated matrix was prepared to evaluate the performance of these markers in all tested *Leishmania* specimens.

Consistency of AFLP profiles was tested using two approaches. First, the reproducibility of the technique was estimated by performing, at one time point, ten full replicates from a single culture of *L. panamensis* promastigotes (Ps reference isolate), from DNA extraction to allele calling using one primer combination (EcoRI-0/MseI-G). The error rate was then estimated between every possible pair of replicates, using previously described procedures [28]–[29]. In a second approach we wanted to examine the stability of AFLP profiles during *in vitro* propagation to check whether this procedure could be responsible for the generation of the genetic variability detected. Promastigotes of the same strain were cultured for one year, under the same culture conditions, subculturing twice a week. Genomic DNA was extracted from samples taken every month and AFLP profiles were generated using ten selective primer combinations. The error rate was then calculated for every possible pairwise comparison between time points using the concatenated dataset. In addition, to check if profiles were accumulating changes over time, pairwise errors were calculated between the dataset generated at the first month and those generated at each subsequent time point. In this time course experiment, the estimation of error rates was done considering two types of band mismatches: “unstable mismatches” (UMM; alleles appearing and disappearing sporadically, possibly representing error of the technique) and “stable mismatches” (SMM; alleles which appear or disappear once and stay that way, representing also putative real new polymorphisms).

### Data Analysis

A concatenated presence/absence matrix was used to score polymorphism levels, count group-specific fixed, private or fixed-private alleles, and derive a Jaccard distance (Jaccard distance = 1 −Jaccard similarity; [30]). This distance matrix was used for phylogenetic and ordination analyses. Individual matrices, containing only presence/absence data from individual selective primer combinations were used to calculate primer-specific Jaccard distance matrices. These individual distance matrices were tested for concordance by means of Mantel tests [31], as implemented in PAST v2.17b software [32]. The test statistic *R* ranges from −1 to 1, and statistical significance was estimated by permutation tests.

Relationships among specimens and taxa were studied using distance based methods, as recommended for dominant, anonymous markers. Phylogenetic relationships were explored using phylogenetic networks [33]. Split graphs depicting phylogenetic relationships among specimens were constructed using Jaccard distance data transformation and the Neighbor-Net method [34], as implemented in SplitsTree 4 v4.12.6 [35]. Robustness of clustering was tested by nonparametric bootstrapping (1000 resamplings). As an additional measure of the consistency of clustering, other clustering methods were applied to the same dataset, namely Bio Neighbor Joining [36] and Unweighted Pair Group Method with Arithmetic mean (UPGMA; [37]) using the same software. Additionally, ordination analyses were performed for a better understanding of the multivariate nature of AFLP data in a lower dimensional space. Principal coordinate analysis (PCoA) was applied to the Jaccard distance matrix using FAMD (Fingerprint Analysis with Missing Data, v 1.25, release May 2010) [38].

## Results

Here we show results of studying the genetic variation in *Leishmania* parasites circulating in Panama as depicted by the anonymous, multilocus fingerprinting technique AFLP. We analyzed samples of parasites isolated from local patients suffering cutaneous leishmaniasis as well as several reference strains covering both subgenera, *Leishmania* (*Viannia*) and *Leishmania* (*Leishmania*). All tested *Leishmania* specimens, which included specimens of *L. panamensis*, *L. guyanensis*, *L. braziliensis*, *L. peruviana*, *L. lainsoni*, *L. chagasi*, *L. major*, *L. mexicana*, *L. aristidesi* and *L. donovani* (Table 1), were successfully typed using *hsp*70 gene PCR – RFLP (data not shown). All *Leishmania* specimens isolated from local patients turned out to be *L. panamensis*.

The implementation of the AFLP, using a rather conservative procedure for allele calling, allowed the generation of a significant number of fragments. In total, 2457 loci were scored from the 42 specimens and 13 selective primers. Out of these 2457 loci, 2455 were polymorphic. Many fixed, private and fixed private fragments were observed for different groups of specimens. The group corresponding to *L. panamensis* specimens presented 1104 fragments, of which 781 were polymorphic, 323 fixed, 294 private and 37 fixed private fragments (Table 2). Although numbers may not be representative due to the small number of specimens tested, the closely related species *L. guyanensis* showed 739 fragments, of which 375 were polymorphic, 364 were fixed, 103 were private, and 21 were fixed private alleles.

**Table 2:** Total number of fragments, polymorphisms and taxon-specific fragments detected by AFLP analysis of all *Leishmania* specimens tested.

				Group specific fixed private alleles (bp)		
Code	Selective primer combination (^1^)	Bands detected in*L. panamensis*	Polymorphism in*L. panamensis* (%)	*L. panamensis*	Subgenus *Viannia*	Subgenus *Leishmania*
R10	EcoRI-0/MseI-A	99	89	159	–	–
R11	EcoRI-0/MseI-C	97	59	180, 312	64, 66, 116, 136, 141, 219, 270, 351, 399	–
R12	EcoRI-0/MseI-G	85	63	65, 183, 200, 341, 355, 373	66, 92, 136, 226, 250	120
R13	EcoRI-0/MseI-T	102	74	156, 356, 389	88, 205, 265	-
S9	EcoRI-A/MseI-0	113	73	367	116, 216, 341	–
S12	EcoRI-A/MseI-G	79	64	67, 141, 154, 239	92, 104, 160, 165	–
S13	EcoRI-A/MseI-T	78	79	377,	216	103
T9	EcoRI-C/MseI-0	107	56	101, 183, 411,	198, 219, 351	121
U9	EcoRI-G/MseI-0	105	77	74, 85, 133, 134, 252	66, 135, 245	131, 170
V9	EcoRI-T/MseI-0	75	49	88, 136, 144, 153, 210, 330	76, 99, 106, 162, 180	–
V13	EcoRI-T/MseI-T	71	78	155, 197, 208	100, 180	–
W13	EcoRI-AA/MseI-T	52	86	–	87, 128, 203	–
Z12	EcoRI-AT/MseI-G	41	70	170, 211	165	–
	Total	1104	70			

(^1^) EcoRI: 5′-GACTGCGTACCAATTC-3′; MseI: 5′-GATGAGTCCTGAGTAA-3′.

The analysis of the *L. panamensis* group revealed varied proportions of polymorphisms, ranging from 49% (primer combination V9) to 89% (primer combination R10, Table 2), and 70% when considering all primers. All *L. panamensis* isolates showed different fingerprints. Several fragments were detected which appeared to be specific for the groups *L. (Viannia), L. (Leishmania)* and *L. panamensis* (Table 2).

The pairwise error values obtained from the reproducibility experiment ranged from 0% (identical profiles) to 6.3% (most divergent profiles), with a mean value of 3.1%. When we checked the stability of AFLP profiles in time, the mean estimated pairwise errors were 2.5%, 0.27%, and 2.2% when considering all mismatches, only “stable mismatches”, or only “unstable mismatches”, respectively (Figure S1, panel A). These figures suggest that most of the mismatches observed are probably due to errors of the procedure, rather than generation of genuine new polymorphisms. When we compared the AFLP profiles from the first month against profiles generated during each subsequent month, the error rate values did not show a significant correlation with time, either when considering “stable” or “unstable” alleles (Figure S1, panels B and C). As error rates did not show a tendency to increase with time (Spearman correlation *P*>0.05 in both cases), it seems that at least in this isolate, and under our experimental conditions, the *in vitro* propagation required for the AFLP procedure does not seem to significantly contribute to the variability observed in this study.

As an additional assessment of the properties of AFLP markers for these species, we evaluated the congruency of distance matrices generated from each particular selective primer combination, using all specimens. Each possible pair of Jaccard distance matrices was compared by means of Mantel tests, which showed that all datasets were strongly and significantly correlated (Mantel R values ranged from 0.88 to 0.98, all highly significant at *P*<0.001; Table S1).

**Figure 1:**
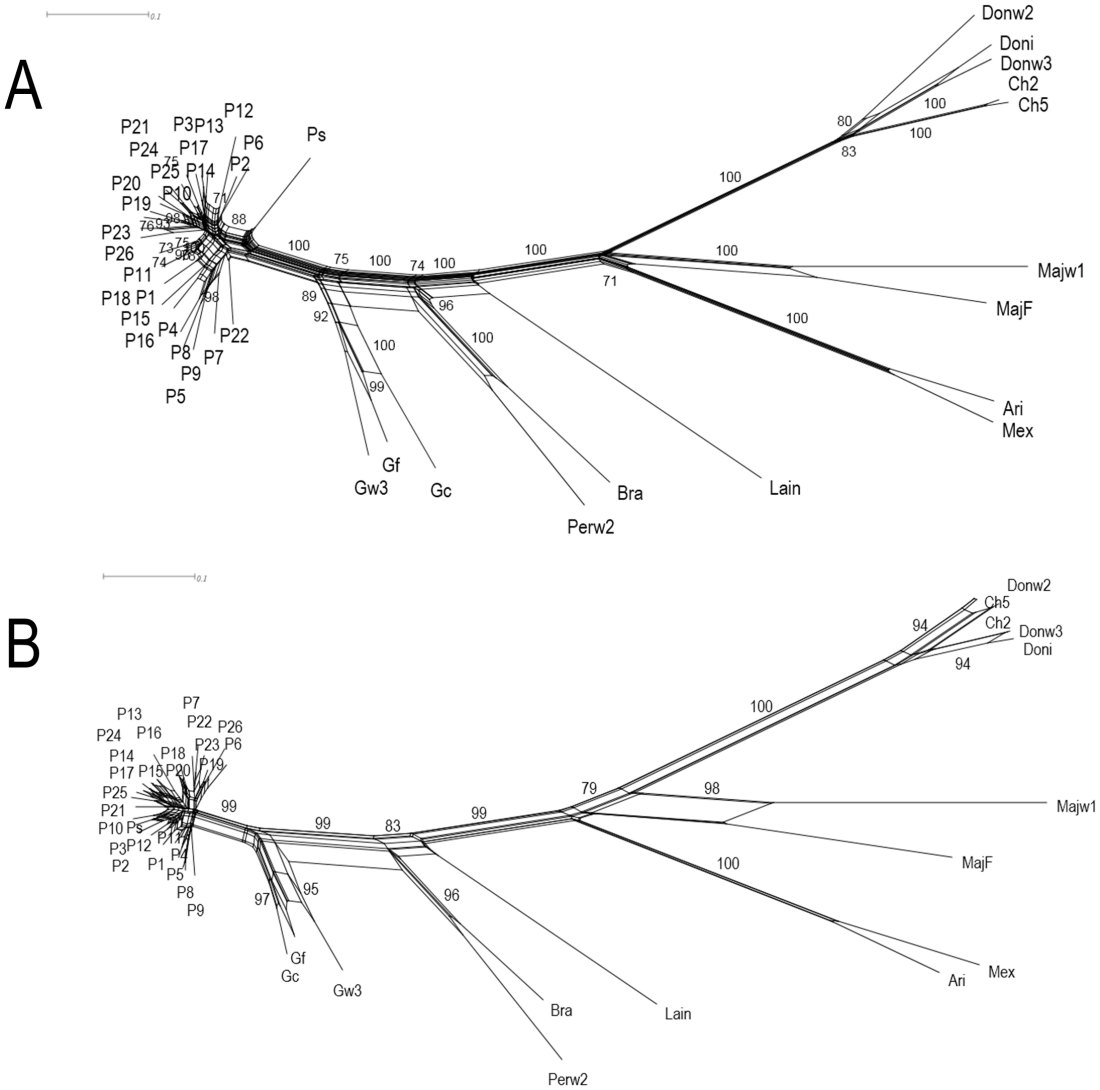
Split graph showing the results of Neighbor-Net analysis obtained on Jaccard distances among *Leishmania* species tested. Specimen names according to Table 1. Bootstrap values over 70% are shown. Panel A: split graph generated using the concatenated matrix (fit: 96%). Panel B: split graph generated using matrix corresponding to selective primer V9 (fit: 97%).

Cluster and ordination analyses were done on concatenated presence – absence matrix to explore the relationships among all tested species of *Leishmania*. The Neighbor-Net split graph obtained after Jaccard transformation showed very good species definition, with very high bootstrap support (Figure 1A). Within the *L. panamensis* group, some groups could also be well defined with significant statistical support. Trees generated using other methods, namely BioNJ and UPGMA, showed the same overall topologies (Figure S2). The Neighbor-Net phylogenetic network displayed perfect definition of species into both subgenera, *Leishmania* (*Viannia*) and *Leishmania* (*Leishmania*). The genetic relationships among the *Leishmania* (*Viannia*) specimens showed clear distinction of all species tested, and even closely related species like *L. panamensis* and *L. guyanensis* could be well separated with strong statistical support. This split graph also showed a significant number of incompatible splits, particularly among *L. panamensis* and *L. guyanensis* isolates. As also shown by several authors from the analyses of other marker systems, *L. peruviana* and *L. braziliensis* occupy closely related but clearly distinct positions, while *L. lainsoni* is represented as the most divergent species within the *L. (Viannia)* subgenus. The *L. (Leishmania)* subgenus group was well defined with robust clusters for the included species. We tested the ability of each individual matrix to allow reconstruction of phylogenetic relationships among all specimens. We detected some selective primer combinations, particularly combination V9 (Table 2), that were able to generate trees with almost similar resolution and topology as the concatenated dataset (Figure 1B). However, using the full concatenated matrix was required to achieve better intra-specific node resolution in *L. panamensis* (Table S2).

**Figure 2:**
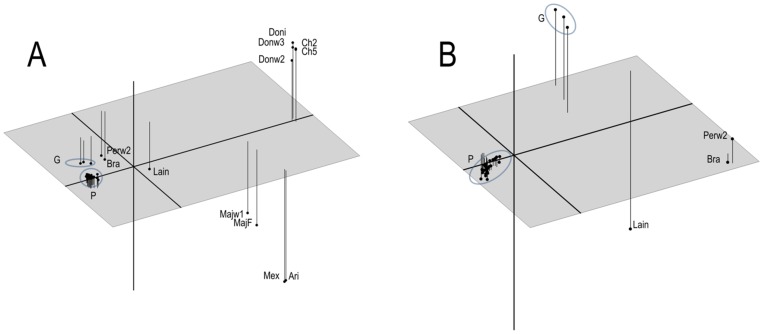
Principal coordinate analysis (PCoA) plots depicting genetic relationships among *Leishmania* species tested. Panel A: PCoA plot including all specimens tested. P, *L. panamensis* strains; G, *L. guyanensis* strains. Other specimen names according to Table 1. The variance explained is 64%. Panel B: PCoA plot including only species from *Leishmania* (*Viannia)* subgenus. The variance explained is 63%.

The principal coordinate analysis confirmed the results of the clustering analyses, showing a clear separation between specimens of the *Leishmania (Viannia)* and *Leishmania (Leishmania)* subgenera, the latter being the most diverse (Figure 2A). When the PCoA plot was done including only the *Leishmania (Viannia)* species, more resolution was observed in three dimensions (Figure 2B), confirming the clear separation between *L. panamensis* and *L. guyanensis*, the *L. peruviana* – *L. braziliensis* relationship, and the divergent position of *L. lainsoni*.

## Discussion

This study presents a genetic analysis of *Leishmania* parasites circulating in Panama as well as several reference strains covering both subgenera, *Leishmania* (*Viannia*) and *Leishmania* (*Leishmania*), using AFLP markers. The AFLP system has been applied frequently in other taxa, and is convenient because of its reproducibility and sequence information independence. Once optimized for a specific taxonomic group, AFLP allows easy and rapid genome screening for polymorphisms, and the detection of hundreds to thousands of variable sites.

Prior to AFLP typing, we tested species identity of all specimens by using *hsp70* PCR-RFLP. All isolates from cutaneous leishmaniasis patients were identified as *L. panamensis*, in agreement with a previous report in Panama [17]. Other species have been sporadically reported in Panama, but their clinical relevance is still to be defined [18].

When we analyzed our specimens with the AFLP procedure, large numbers of fragments could be detected in all cases. Of particular interest were the fixed private alleles which, if validated with more isolates, may represent diagnostic fragments useful for rapid molecular species discrimination in a clinical setting. These fixed private alleles are particularly interesting because the *Leishmania* species that have been sequenced so far have a very high level of synteny sharing and a very low number of species-specific genes [39].

In terms of the number of fragments, our findings differ from those reported by Kumar and coworkers in *Leishmania donovani* [21]. Employing the same restriction enzyme system (Tru9I – EcoRI, and +3 primers), they found high number of fragments, at levels we observed in *L. panamensis* only when using lower stringency primers (+0, +1, +2). Although not directly comparable to those of Kumar et al., our results are congruent with the fact that the *Leishmania* genome is GC rich. Therefore, the use of these restriction enzymes should generate fewer fragments in the useful range of 50 to 500 base pairs [27].

The results of the AFLP typing revealed very high levels of polymorphisms, especially in *L. panamensis*. Odiwuor and colleagues [23] employed a different combination of enzymes (TaqI – PstI) to analyze the genetic diversity in *L. archibaldi*, *L. donovani*, *L. chagasi* and *L. infantum*. They found that up to 52% of alleles were polymorphic, a level of variability that is high enough to describe the genetic variability of the tested species and strains by means of clustering and ordination techniques. In a more recent report, these authors used the same AFLP system to analyze some species of *Leishmania (Viannia)* [24]. The proportions of polymorphisms that we observed in *L. panamensis* are even higher than those reported for other *Leishmania* species in these studies. This might be because more strains were typed in our study, and/or because some of the selective primer combinations that we used might probe regions of the *L. panamensis* genome that are more polymorphic.

Several molecular events may result in an AFLP fragment polymorphism, including mutations at any of the restriction sites, deletions, insertions, translocations, or variation in the size of repetitive sequences. Genomic DNA rearrangements at repetitive sequences have been shown to be rather common in protozoa, contributing to their high genetic variability [40]–[41]. It has been shown that *Leishmania* genomic DNA may contain up to 25% diverse repetitive sequences, as judged from DNA reassociation kinetics in *L. donovani* [42]. More recently, genome sequencing has shown that one member of the *Leishmania (Viannia)* subgenus, *L. braziliensis*, contains a higher number of repetitive sequences than either *L. major* or *L. infantum*, as well as more divergence at the level of DNA and protein sequences [43]. As some of these repetitive sequences are intrinsically polymorphic, they may be contributing to the AFLP polymorphisms that we observed in *L. panamensis*.

Although AFLP is considered to be a very reproducible fingerprinting technique [44], it is not free of potential errors, which in turn can have a significant effect on further analyses [28]. In our experimental conditions, AFLP had good reproducibility, with error rates roughly similar to values reported by other authors in other organisms [28]. The time stability experiment showed that, at least under our experimental conditions and using this strain, AFLP profiles appear to be stable in time. This is in agreement with the general belief that *Leishmania* karyotypes are stable *in vitro* [45]. Our result, however, might be species and/or strain specific, as it has been reported that some strains of *L. peruviana* may undergo significant karyotype changes during *in vitro* propagation [46].

The AFLP data presented here also provide some insight into the reproductive mode of these parasites. The Jaccard distance matrices generated by each individual selective primer pair combination (which may be regarded as independent markers), were strongly correlated when all *Leishmania* specimens were analyzed together. This suggests some degree of linkage disequilibrium, and is consistent with a predominantly clonal mode of propagation. Similar levels of correlation between independent molecular markers have been observed in *Trypanosoma cruzi,* and interpreted as evidence of a predominantly clonal mode of reproduction [47]. In this regard, our data are consistent with previously reported evidence for the genus [48]. However, this mode of reproduction would also lead one to predict the presence of frequent, repeated fingerprint profiles in the parasite populations. This prediction was not confirmed in our study, as all isolates of *L. panamensis* had different AFLP profiles. Possible causes for this discrepancy may include: an insufficient sample size, sporadic but significant sexual reproduction, a more heterogeneous range of sand fly vectors and/or hosts than previously suspected for this geographical area, and/or more complex transmission cycles. Studies in *L. braziliensis* using other markers have found higher molecular diversity in areas with more sylvatic associated transmission cycles [49]. In Panama, accelerated urbanization frequently disrupts natural environments, possibly allowing for more sylvatic related transmission cycles, and therefore higher molecular diversity.

The AFLP data obtained from all tested parasite species allowed us to reconstruct phylogenetic networks that were congruent with the accepted taxonomy for the genus [50]–[53]. Within the *L. panamensis* group, split graphs showed a considerable amount of reticulation, possibly indicating sampling artifacts or some levels of genetic recombination. Interestingly, some individual selective primer combinations were able to produce networks which robustly reproduced the accepted taxonomy for all specimens. This result is important because performing AFLP using single selective primer combinations should be more affordable and convenient.

The clear separation observed between the closely related *L. panamensis* and *L. guyanensis* (particularly in the principal coordinate plot), is not consistent with previous reports questioning the validity of the separation of these two species based on MLEE and RAPD data [54]–[55]. The higher resolution observed in our results might be due to the fact that AFLP allows simultaneous examination of thousands of loci in the *Leishmania* genome, most of them polymorphic. Although the trees generated with our data show topologies concordant with previous phylogenetic studies for the genus *Leishmania*, confirmation of the usefulness of AFLP data may require inclusion of more strains, especially for species represented here by a single specimen.

The results of our phylogenetic and ordination analyses indicate that AFLP markers are a useful tool for studying the genetic diversity of the *Leishmania* genus at a higher resolution than was possible with previously used markers. Additionally, AFLP scanning of *Leishmania* genomes should allow for the rapid identification of polymorphisms associated with clinically relevant traits, such as drug resistance or clinical presentation. Rapid conversion of those polymorphisms into dominant markers would have an immediate application to the clinical practice.

Although the AFLP approach has some limitations associated with the dominant mode of inheritance and the requirement of purified DNA, the possibility of simultaneously examining hundreds or even thousands of sites in the genome is an attractive opportunity to study genetic variation at depths only possible today with next generation sequencing, but at much higher costs.

Here we have demonstrated that AFLP markers allow high resolution genetic analysis of parasites of the *Leishmania* genus, particularly of the isolates circulating in Panama. We have uncovered a high number of polymorphisms in the main species causing cutaneous leishmaniasis, *L. panamensis*. This discovery opens up new and exciting possibilities for the generation of knowledge in the fields of molecular epidemiology and taxonomy, and confirms the notion that AFLP is a very promising tool for studying the genetic diversity of these parasites.

## Supporting Information

Figure S1 Characterization of mismatches (error rate) detected in AFLP profiles during *in vitro* cultivation of promastigotes of the *Leishmania panamensis* Ps isolate during one year. Panel A: box plot showing distribution of pairwise errors (see Materials and Methods for details) considering all mismatches (allMM), stable mismatches (SMM) or unstable mismatches (UMM). Panels B and C: pairwise error rates (considering all mismatches or stable mismatches, respectively) between profiles generated at each time point and the one obtained at first month.
(TIF)

Figure S2 Split graphs obtained from Jaccard distance transformations of the concatenated AFLP matrix for all *Leishmania* specimens tested, following the algorithms Bio Neighbor Joining (A) and UPGMA (B).
(TIF)

Table S1 Pairwise Mantel test for Jaccard distance matrices generated from each selective primer combination.
(DOCX)

Table S2 Number of significant nodes and bootstrap values of UPGMA trees generated from datasets obtained from each selective primer combination. Bootstrap values were calculated after 10 000 resamplings, and only values over 70% are showed.
(DOCX)

Methods S1 Details of AFLP data analysis in GeneMarker v 2.2.0 (SoftGenetics LLC, USA).
(DOCX)
