# Supplementary figures and images for: AFLP Polymorphisms Allow High Resolution Genetic Analysis of American Tegumentary Leishmaniasis Agents Circulating in Panama and Other Members of the Leishmania Genus
Source: PLoS One. 2013 Sep 9;8(9):e73177. doi: 10.1371/journal.pone.0073177 (PMC3767818; doi:10.1371/journal.pone.0073177)

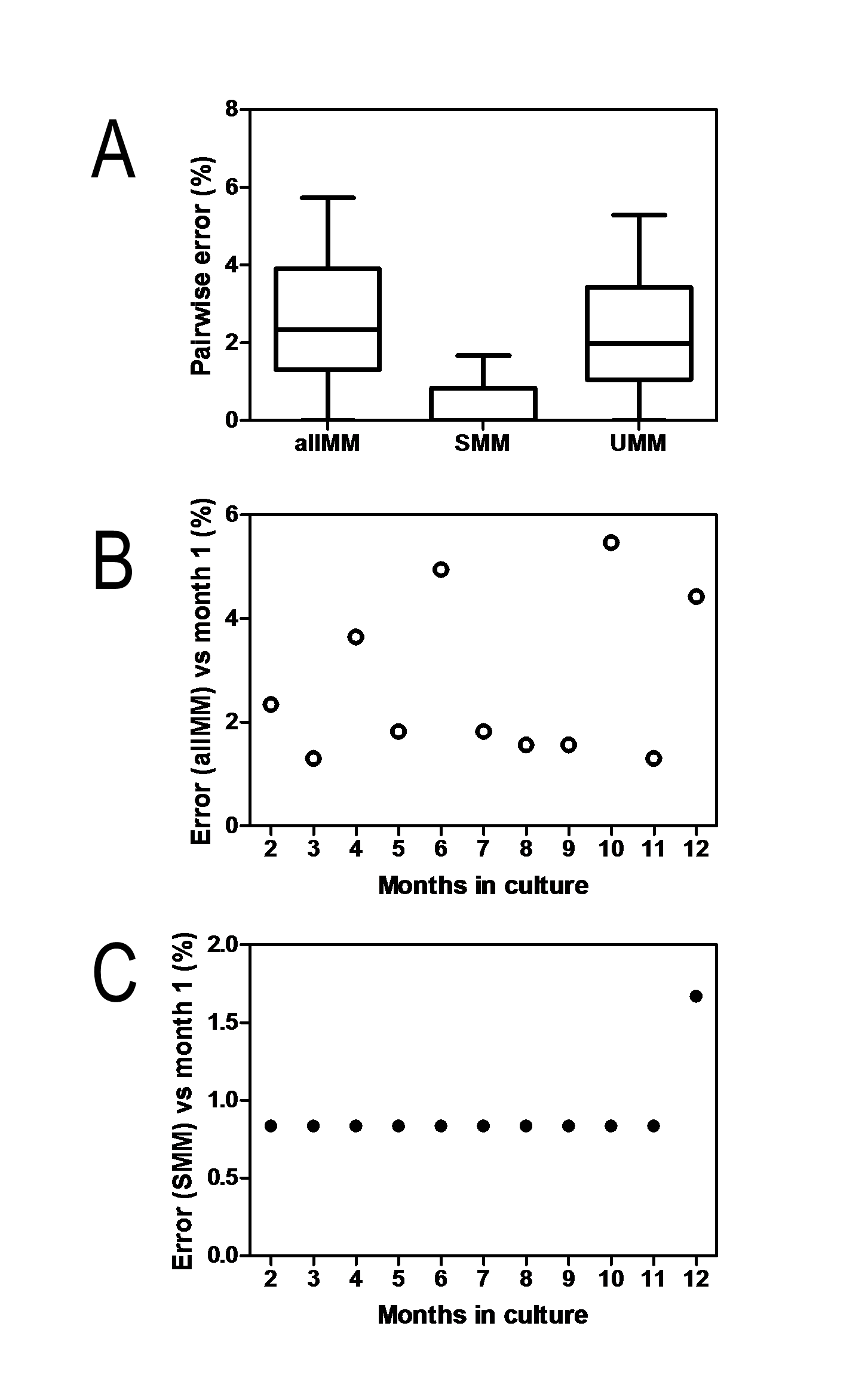

Supplement: Figure S1 — Characterization of mismatches (error rate) detected in AFLP profiles during in vitro cultivation of promastigotes of the Leishmania panamensis Ps isolate during one year. Panel A: box plot showing distribution of pairwise errors (see Materials and Methods for details) considering all mismatches (allMM), stable mismatches (SMM) or unstable mismatches (UMM). Panels B and C: pairwise error rates (considering all mismatches or stable mismatches, respectively) between profiles generated at each time point and the one obtained at first month. (TIF) [file pone.0073177.s001.tif]

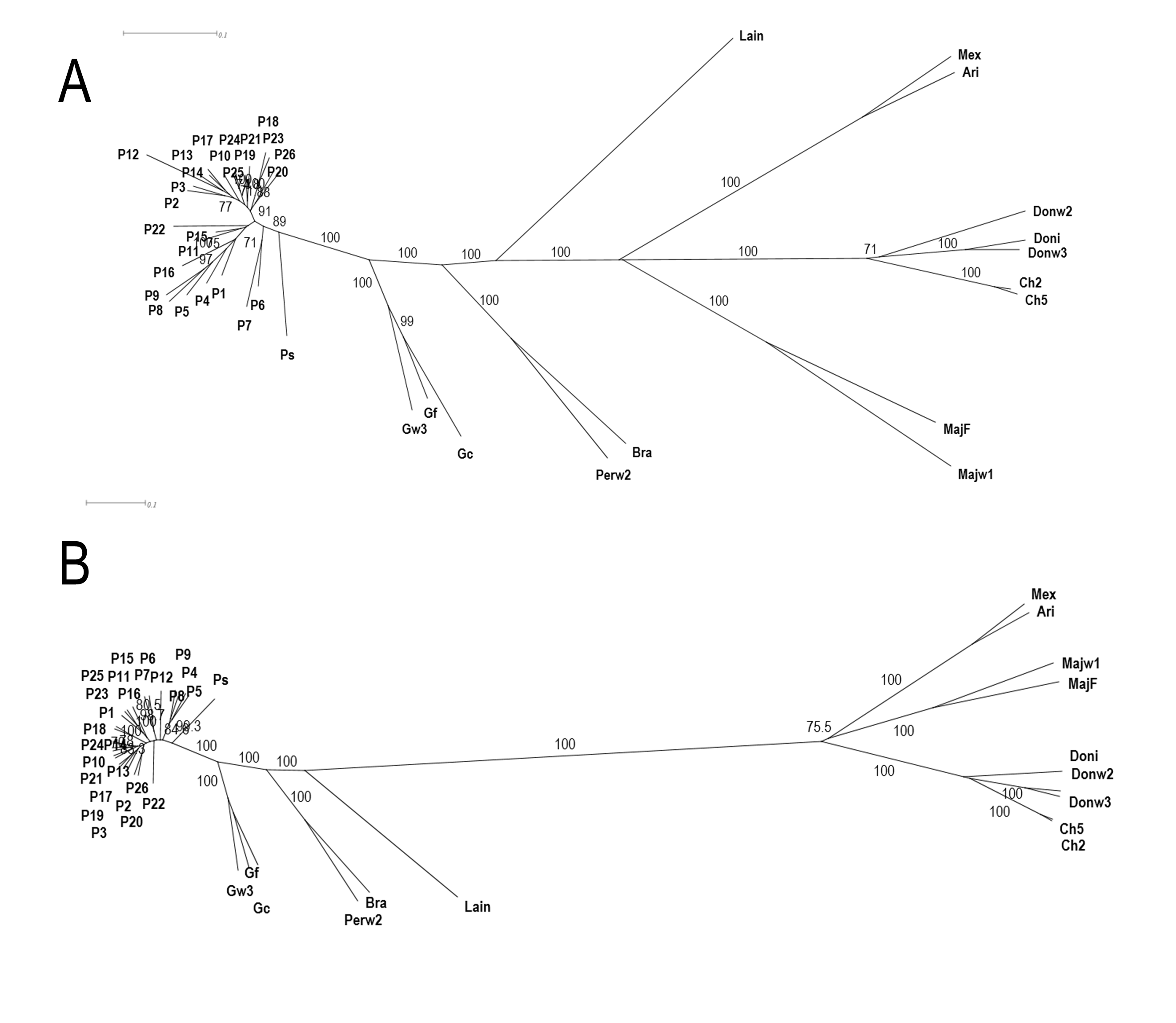

Supplement: Figure S2 — Split graphs obtained from Jaccard distance transformations of the concatenated AFLP matrix for all Leishmania specimens tested, following the algorithms Bio Neighbor Joining (A) and UPGMA (B). (TIF) [file pone.0073177.s002.tif]
