# Supplementary material for: AFLP Polymorphisms Allow High Resolution Genetic Analysis of American Tegumentary Leishmaniasis Agents Circulating in Panama and Other Members of the Leishmania Genus
Source: PLoS One. 2013 Sep 9;8(9):e73177. doi: 10.1371/journal.pone.0073177 (PMC3767818; doi:10.1371/journal.pone.0073177)
Supplement: Table S1 — Pairwise Mantel test for Jaccard distance matrices generated from each selective primer combination. (DOCX) [file pone.0073177.s003.docx]

**Supporting Information for:**

AFLP Polymorphisms Allow High Resolution Genetic Analysis of American Tegumentary Leishmaniasis Agents Circulating in Panama and other Members of the *Leishmania* Genus.

Carlos M. Restrepo, Carolina De La Guardia, Octavio E. Sousa, José E. Calzada, Patricia L. Fernández, Ricardo Lleonart.

Supplementary Table S1

Table S1. Pairwise Mantel test for Jaccard distance matrices generated from each selective primer combination.

| Selective primer combination | Pairwise *R* values | | | | | | | | | | | | |
| --- | --- | --- | --- | --- | --- | --- | --- | --- | --- | --- | --- | --- | --- |
|  | R10 | R11 | R12 | R13 | S9 | S12 | S13 | T9 | U9 | V9 | V13 | W13 | Z12 |
| R10 | 1 | 0.9261 | 0.9267 | 0.9188 | 0.9087 | 0.9168 | 0.9088 | 0.9246 | 0.8859 | 0.9106 | 0.9307 | 0.9174 | 0.902 |
| R11 |  | 1 | 0.9889 | 0.9711 | 0.9766 | 0.9794 | 0.975 | 0.9873 | 0.9642 | 0.9822 | 0.9727 | 0.9265 | 0.964 |
| R12 |  |  | 1 | 0.9697 | 0.9739 | 0.979 | 0.9748 | 0.9885 | 0.9612 | 0.9825 | 0.9744 | 0.9314 | 0.9588 |
| R13 |  |  |  | 1 | 0.9515 | 0.9544 | 0.958 | 0.9616 | 0.9506 | 0.9668 | 0.9663 | 0.9151 | 0.9505 |
| S9 |  |  |  |  | 1 | 0.9659 | 0.9667 | 0.9698 | 0.9584 | 0.9622 | 0.9582 | 0.9137 | 0.9437 |
| S12 |  |  |  |  |  | 1 | 0.9679 | 0.9782 | 0.9471 | 0.9729 | 0.9614 | 0.922 | 0.9583 |
| S13 |  |  |  |  |  |  | 1 | 0.9696 | 0.9627 | 0.9635 | 0.961 | 0.9115 | 0.954 |
| T9 |  |  |  |  |  |  |  | 1 | 0.9592 | 0.984 | 0.973 | 0.9174 | 0.9647 |
| U9 |  |  |  |  |  |  |  |  | 1 | 0.944 | 0.9498 | 0.8848 | 0.9565 |
| V9 |  |  |  |  |  |  |  |  |  | 1 | 0.9708 | 0.9187 | 0.9603 |
| V13 |  |  |  |  |  |  |  |  |  |  | 1 | 0.9281 | 0.9538 |
| W13 |  |  |  |  |  |  |  |  |  |  |  | 1 | 0.8966 |
| Z12 |  |  |  |  |  |  |  |  |  |  |  |  | 1 |

Above diagonal: Mantel test *R* value, based on Jaccard distances (1 – Jaccard similarities), significance based on 10 000 permutations. All *R* values were statistically significant (*P* < 0.001, one tailed).
