# Supplementary material for: AFLP Polymorphisms Allow High Resolution Genetic Analysis of American Tegumentary Leishmaniasis Agents Circulating in Panama and Other Members of the Leishmania Genus
Source: PLoS One. 2013 Sep 9;8(9):e73177. doi: 10.1371/journal.pone.0073177 (PMC3767818; doi:10.1371/journal.pone.0073177)
Supplement: Table S2 — Number of significant nodes and bootstrap values of UPGMA trees generated from datasets obtained from each selective primer combination. Bootstrap values were calculated after 10 000 resamplings, and only values over 70% are showed. (DOCX) [file pone.0073177.s004.docx]

**Supporting Information for:**

AFLP Polymorphisms Allow High Resolution Genetic Analysis of American Tegumentary Leishmaniasis Agents Circulating in Panama and other Members of the *Leishmania* Genus.

Carlos M. Restrepo, Carolina De La Guardia, Octavio E. Sousa, José E. Calzada, Patricia L. Fernández, Ricardo Lleonart.

Supporting Table S2:

Table S2. Number of significant nodes and bootstrap values of UPGMA trees generated from datasets obtained from each selective primer combination. Bootstrap values were calculated after 10 000 resamplings, and only values over 70% are showed.

| Selective primer combination | Number of nodes detected with bootstrap values over 70% | Bootstrap values |
| --- | --- | --- |
| R10 | 17 | 100, 87, 100, 100, 78, 100, 99, 70, 77, 78, 93, 96, 99, 98, 89, 99, 87 |
| R11 | 16 | 76, 98, 100, 100, 81, 94, 99, 100, 99, 99, 99, 83, 99, 99, 95, 73 |
| R12 | 17 | 81, 100, 100, 100, 99, 100, 100, 99, 92, 97, 99, 99, 99,85, 72, 79, 79 |
| R13 | 15 | 100, 75, 99, 100, 100, 73, 89, 99, 99, 72, 97, 88, 75, 88, 98 |
| S9 | 14 | 92, 99, 100, 92, 81, 100, 94, 91, 100, 100, 98, 89, 98, 81 |
| S12 | 13 | 93, 99, 100, 100, 95, 99, 99, 73, 84, 100, 94, 99, 91 |
| S13 | 18 | 100, 74, 99, 100, 100, 71, 93, 99, 94, 95, 98, 99, 90, 97, 77, 88, 94, 75 |
| T9 | 19 | 100, 98, 100, 100, 99, 100, 95, 99, 93, 98, 92, 99, 100, 74, 92, 81, 91, 76, 97 |
| U9 | 15 | 100, 100, 97, 94, 98, 79, 100, 99, 99, 99, 100, 100, 100, 100, 95 |
| V9 | 14 | 100, 100, 99, 83, 99, 96, 97, 87, 83, 99, 85, 88, 99, 83 |
| V13 | 13 | 100, 79, 87, 98, 74,93, 98, 86, 96, 98, 82, 71, 80 |
| W13 | 14 | 79, 100, 74, 77, 92, 77, 99, 99, 93, 77, 98, 70, 87, 82 |
| Z12 | 7 | 94, 92, 87, 100, 91, 99, 78 |
| Concatenated dataset | 22 | 100, 75, 98, 100, 100, 100, 100, 100, 100, 100, 100, 100, 100, 100, 98, 77, 82, 99, 99, 83, 99, 81 |
