# Supplementary material for: AFLP Polymorphisms Allow High Resolution Genetic Analysis of American Tegumentary Leishmaniasis Agents Circulating in Panama and Other Members of the Leishmania Genus
Source: PLoS One. 2013 Sep 9;8(9):e73177. doi: 10.1371/journal.pone.0073177 (PMC3767818; doi:10.1371/journal.pone.0073177)
Supplement: Methods S1 — Details of AFLP data analysis in GeneMarker v 2.2.0 (SoftGenetics LLC, USA). (DOCX) [file pone.0073177.s005.docx]

**Supporting Information for:**

AFLP Polymorphisms Allow High Resolution Genetic Analysis of American Tegumentary Leishmaniasis Agents Circulating in Panama and other Members of the *Leishmania* Genus.

Carlos M. Restrepo, Carolina De La Guardia, Octavio E. Sousa, José E. Calzada, Patricia L. Fernández, Ricardo Lleonart.

**Details of AFLP data analysis in GeneMarker v 2.2.0 (SoftGenetics LLC, USA):**

Details of AFLP protocol followed, as described by the manufacturer, are available online at:

http://www3.appliedbiosystems.com/cms/groups/applied_markets_support/documents/generaldocuments/cms_040935.pdf

Accessed 20 June 2013

Details of AFLP data analysis in GeneMarker v 2.2.0 (SoftGenetics LLC, USA):

**Raw Data Analysis:**

Auto Range (frame)-check: start=0; End= 8971

Peak Saturation-selected

Baseline Substraction-selected

Pull-up correction-selected

Spike removal-selected

Size call: Local Southern-selected

**Allele Call:**

Start=50; End= 500

Peak detection threshold

Min intensity=100; Max intensity= 30000

Percentage>1 Global Max

Local region %>1 Local max

Stutter peak filter (%)-selected: Left=5; right=5

Plus-A filter-selected

**Allele evaluation:**

Peak score:

Reject<1 check<7<pass

AFLP unconfidence at right side: score<30-selected

AFLP Normalization-selected

**Panel:**

Bin width: 0.5bp right and left

Additional manual check of electropherograms:

In addition to the automatic assignment of alleles by the software, all electropherograms were manually evaluated in order to confirm or discard alleles that were left in check status by the software. Also, some peaks that evidently had the characteristics of a true allele were not assigned by the software and had to be added manually to the presence-absence matrix. Peaks that were identified as alleles but failed to be correctly assigned to a given bin were manually added to the panel in the final Excel matrix. Some alleles that had poor quality in some individuals but were correctly assigned in other close related ones were validated because it was noticeable that they were common alleles with probably amplification problems. All extra validated alleles were at least twice as high as the noise level for each particular electropherogram. The final matrix was edited in Microsoft Excel as a starting point for conversion into the different input formats required by other data analysis softwares.
